# All solution-processed micro-structured flexible electrodes for low-cost light-emitting pressure sensors fabrication

**DOI:** 10.1038/s41598-017-07284-8

**Published:** 2017-07-31

**Authors:** Rie Shimotsu, Takahiro Takumi, Varun Vohra

**Affiliations:** Department of Engineering Science, University of Electro-Communications, 1-5-1 Chofugaoka, Chofu, Tokyo 182-8585 Japan

## Abstract

Recent studies have demonstrated the advantage of developing pressure-sensitive devices with light-emitting properties for direct visualization of pressure distribution, potential application to next generation touch panels and human-machine interfaces. To ensure that this technology is available to everyone, its production cost should be kept as low as possible. Here, simple device concepts, namely, pressure sensitive flexible hybrid electrodes and OLED architecture, are used to produce low-cost resistive or light-emitting pressure sensors. Additionally, integrating solution-processed self-assembled micro-structures into the flexible hybrid electrodes composed of an elastomer and conductive materials results in enhanced device performances either in terms of pressure or spatial distribution sensitivity. For instance, based on the pressure applied, the measured values for the resistances of pressure sensors range from a few MΩ down to 500 Ω. On the other hand, unlike their evaporated equivalents, the combination of solution-processed flexible electrodes with an inverted OLED architectures display bright green emission when a pressure over 200 kPa is applied. At a bias of 3 V, their luminance can be tuned by applying a higher pressure of 500 kPa. Consequently, features such as fingernails and fingertips can be clearly distinguished from one another in these long-lasting low-cost devices.

## Introduction

Since their introduction at the end of the last century, organic semiconductors and organic electronic devices have demonstrated their potential as low-cost alternatives to the state-of-the-art inorganic devices^1–4^. Among these organic devices, a particular attention has been given to organic light-emitting diodes (OLEDs) for two main reasons. One would be that they provide the means to develop next generation display technologies (e.g. flexible wearable displays). Additionally, they provide the possibility to extremely decrease their fabrication cost using roll-to-roll-compatible high productivity processes^1, 5–7^. Both these aspects are related to their capacity to avoid major damages when mechanical stress (e.g. bending or stretching) is applied to organic electronic devices. While small molecules lead to higher device performances, semiconducting polymers provide much higher mechanical properties due to polymer chain entanglement. Furthermore, as they can be deposited through solution process, the fabrication cost of polymer electronics is lower than that of evaporated small molecule systems^8, 9^.

Among the polymer semiconductors, poly(9,9′-dioctylfluorene-co-benzo-thiadiazole) (F8BT) is now considered as one of the state-of-the-art materials used as emitting layers in OLEDs due to the high external quantum efficiencies (EQEs) and luminance efficiencies obtained for F8BT-based devices^10, 11^. While the EQEs of F8BT OLEDs are usually under 10% for conventional device architectures (bottom transparent anode/top metal cathode), recent studies have demonstrated that higher EQEs can be obtained for inverted device architectures using zinc oxide (ZnO) covered indium tin oxide (ITO) as bottom transparent cathode^12^. Organic electronic devices also present the major advantage of being easily integrated with other electronic circuits and devices to produce new and original concepts such as light-emitting pressure sensors with potential applications as user-interactive electronic skin for instantaneous pressure visualization^13^. In fact, in their study, Wang *et al*. demonstrated that, by combining a thin-film-transistor (TFT) active matrix backplane with arrays of OLEDs covered with pressure-sensitive rubber, devices which switch on only when and where pressure is applied can be produced. However, the complicated device architecture presented in their work relies on a multiple-step process which includes micro-patterning and thermal vacuum evaporation resulting in a large increase of the production cost. A device with similar properties (color changing upon application of pressure) was recently introduced by Chou *et al*. in which an electrochromic device is associated with a pressure sensitive tactile sensor^14^. Unlike the TFT-OLEDs-pressure-sensitive rubber combination; the spatial distribution in this second study is obtained through the integration of photolithographic pyramid-like features on a flexible carbon nanotubes-covered polydimethylsiloxane (PDMS) electrode. By applying pressure on the flexible electrode, electrical contact is generated locally which induces a change in the apparent color of the electrochromic material. This approach utilizes a much simpler device architecture compared to the concept presented by Wang *et al*. However, the electrochromic pressure-sensitive devices require a relatively long color-switching time (largely over 1 s) and no bright emission can be observed from the devices (they cannot be used in the dark).

Here, we present our approach to generate light-emitting pressure sensors using low-cost solution processes. The simple device architecture that we introduce consists of an inverted OLED device in which the top anode, which is commonly deposited by thermal evaporation, is replaced by a hybrid flexible and pressure sensitive electrode composed of PDMS, poly(3,4-ethylenedioxythiophene):polystyrene sulfonate (PEDOT:PSS) and silver nanowires (AgNWs). Furthermore, using self-assembled templates, we produce micro-structured PDMS-based flexible electrodes, which induce enhanced pressure sensitivity and provide a higher spatial resolution as compared to the flat flexible electrodes. The flexible PDMS-based electrodes are first tested in resistive pressure sensors. Compared to the evaporated electrodes, the solution-processed ones exhibit a higher transmittance and introduce the possibility to obtain pressure-sensitive pressure sensing. When these electrodes are used as anode in inverted OLED architectures, our all-solution-processed devices prepared using scalable and low-cost fabrication techniques exhibit strong luminance at relatively low voltages and consequently, open the way to the next generation of interactive electronic skins and pressure sensors.

## Results and Discussion

### Solution-processed PDMS-based electrodes

Previous studies on PDMS-AgNWs hybrid flexible electrodes have demonstrated that deposition of AgNWs on PDMS substrates can be rather problematic due to the low wetting of this inorganic elastomer by the AgNWs solution^15^. In fact, to obtain highly conductive hybrid electrodes, Huang *et al*. used a combination of two processes, namely, the pre-straining/post-embedding process and the spraying of the AgNWs. However, both these processes lack control and cannot be easily scaled up to generate large-scale devices. On the other hand, Chen *et al*. proposed to use alternative materials such as polyethylene terephthalate (PET) as flexible substrates to deposit AgNWs/PEDOT:PSS hybrid electrodes which could be used in capacitive pressure sensors with the following device architecture: PET/AgNWs + PEDOT:PSS/PDMS/AgNWs + PEDOT:PSS/PET^16^. In these capacitive pressure sensors, a PDMS layer placed between the two PET-based flexible electrodes is needed to provide pressure sensitivity. Consequently, these flexible electrodes cannot be readily used for light-emitting pressure sensors. In this section, we will demonstrate that by using an additional PEDOT:PSS compatibilizing layer and integrating micro-structures on the surface of the PDMS substrates, we can achieve similar results without the insertion of the interelectrode PDMS layer.

Materials used as electrodes in organic electronics typically have resistivities below 20 Ω.cm. However, as charge injection is limited by the intrinsic charge transport properties of the semiconducting polymers, it is safe to assume that electrodes with higher resistivity values (below 1 kΩ.cm) would still efficiently work in organic devices. The resistivity of AgNWs electrodes are closely related to the wettability of the AgNWs solution on the used substrates. A high wettability typically results in a homogeneous and dense AgNWs network. On the other hand, if the AgNWs dispersion does not spread well on the surface of the substrate, large aggregates, which considerably reduce the long range percolation ability of the electrodes, will be generated. While direct deposition of the AgNWs suspension in isopropanol (IPA) does not result in homogeneously covered PDMS surfaces, depositing dense AgNWs networks on a PEDOT:PSS layer either by spin-coating or dip-coating has been previously demonstrated^17, 18^. Furthermore, in our recent study, we used solvent additives in the PEDOT:PSS suspension and pre-deposition plasma treatment of the PDMS surface to produce PDMS substrates homogeneously covered with PEDOT:PSS^19^. We compared the performances of PDMS-based flexible electrodes by measuring their resistance at various pressures in devices containing an indium thin oxide (ITO) counter-electrode (Figure S1, Supplementary Information). The compared samples are listed below:

- PDMS/AgNWs (drop-casted)
- PDMS/PEDOT:PSS/AgNWs (drop-casted)
- PDMS/PEDOT:PSS/AgNWs (drop-casted)/PEDOT:PSS
- PDMS/Ag (evaporated reference electrode)

Note that the PEDOT:PSS layer were deposited by spin-coating and all contain a surfactant acting as a wetting agent and dimethyl sulfoxide (DMSO) to improve its conductivity while AgNWs were drop-casted from a dilute solution^20, 21^. Table 1 summarizes the results obtained for the various samples at different applied pressure.

**Table 1:** Measured resistance of PDMS-based flexible electrodes at various pressure.

Flexible electrode type	0 kPa	50 kPa	200 kPa	500 kPa
PDMS/AgNWs	OL^*^	48 MΩ	49 MΩ	47 MΩ
PDMS/PEDOT:PSS/AgNWs	OL^*﻿^	733 Ω	747 Ω	769 Ω
PDMS/PEDOT:PSS/AgNWs/PEDOT:PSS	OL^*﻿^	368 Ω	450 Ω	388 Ω
PDMS/evaporated Ag	OL^*^	<0.1 Ω^*^	<0.1 Ω^*﻿^	<0.1 Ω^*^

^*^OL (Overload) and <0.1 Ω correspond to the upper and lower limits of the measuring equipment, respectively.

From the results presented in Table 1, we can clearly observe that depositing a PEDOT:PSS layer prior to AgNWs results in a large decrease in resistivity of the flexible electrodes, which can be associated with a more homogeneous coverage of the substrate by the AgNWs. Independently of the applied pressure, the resistivity of the flexible electrodes is reduced by a factor of approximately 2 orders of magnitude upon insertion of the PEDOT:PSS interlayer. Note that the variation observed for different pressure is most likely due to measurement errors. By depositing an additional layer of PEDOT:PSS on the PDMS/PEDOT:PSS/AgNWs electrodes, their resistance can be decreased by a factor of 2. While the first PEDOT:PSS layer in contact with PDMS acts as a compatibilizing layer, the second PEDOT:PSS layer deposited on top of the AgNWs decreases the resistance by filling the gaps between adjacent AgNWs but also increases the mechanical stability of these electrodes and modifies their work function so that they can be used as anodes in inverted OLED architectures. Although the solvent-processed electrodes have a lower conductivity compared to that of evaporated Ag ones, the resistance achieved (approx. 400 Ω) should be sufficient to use them as electrodes in organic electronic devices. Additionally, their enhanced mechanical stability compared to evaporated Ag electrodes represents a major advantage as PEDOT:PSS/AgNWs/PEDOT:PSS hybrid electrodes can undergo over 300 repeating cycles (500 kPa pressure ON/OFF) without major changes in the measured resistance, while evaporated Ag flexible electrodes stop functioning after approximately 50 repeating cycles. The rapid deterioration of the evaporated Ag devices is due to the appearance of cracks upon application of mechanical stress through pressure but also to a partial transfer of the evaporated Ag to the counter-electrode bottom substrate. The surface free energy of the ITO counter-electrode is of approximately 60 mJ/m^2^, while those of Ag, PDMS and PEDOT:PSS are 1250, 19.9 and 73 mJ/m^2^, respectively^22–25^. Consequently, Ag has a higher adhesion to the material with the highest surface free energy, namely, ITO and PEDOT:PSS when evaporated and solution-processed electrodes are used, respectively. Lastly, evaporated Ag flexible electrodes have a very low transmittance over the visible range. On the other hand, the hybrid PDMS/PEDOT:PSS/AgNWs/PEDOT:PSS multilayer electrodes have a transmittance of approximately 80% from 350 nm up to 750 nm (Figure S2, Supplementary Information) allowing them to be used for applications such as touch panels.

**Figure 1:**
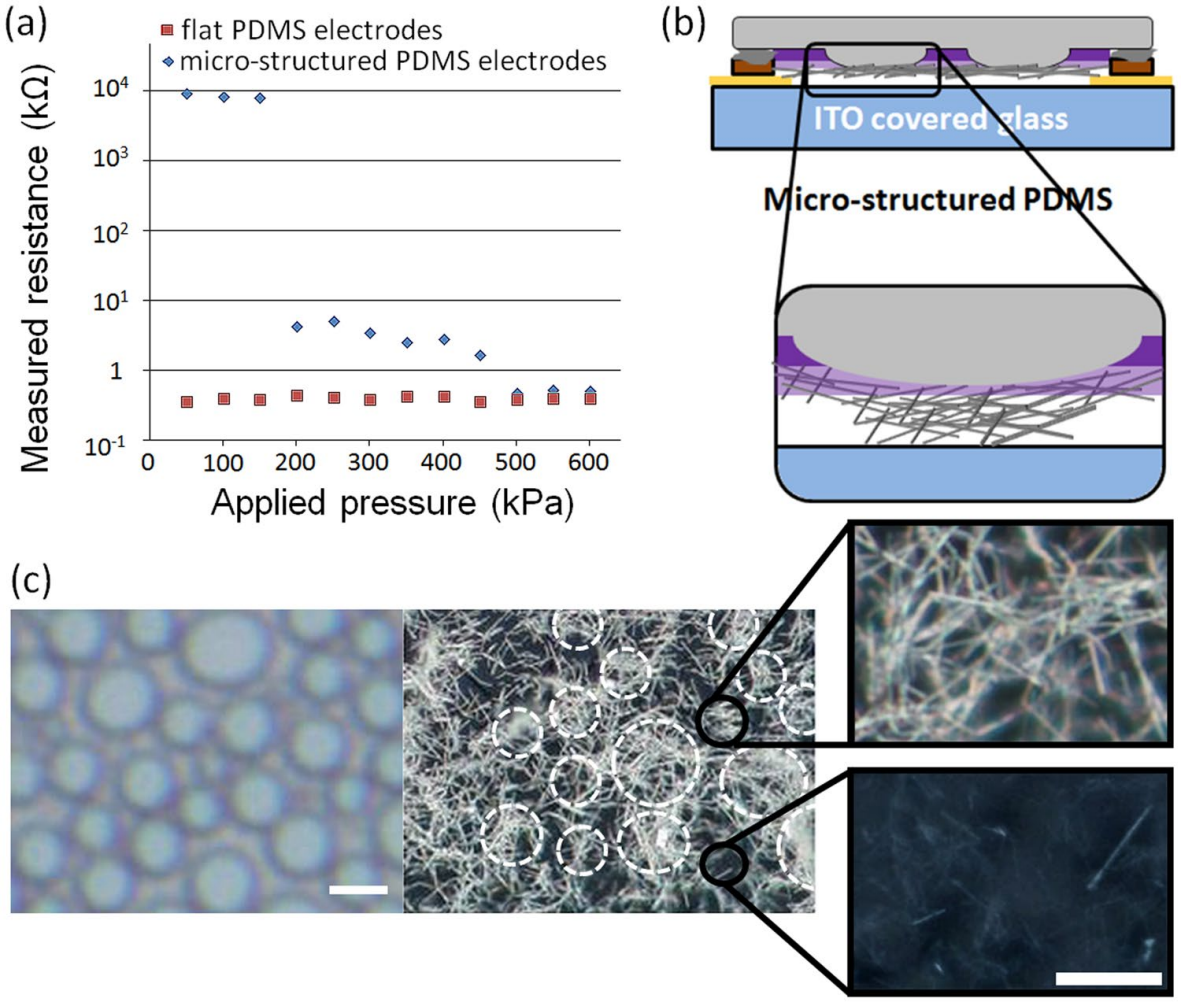
(**a**) Pressure sensitive response of PEDOT:PSS/AgNWs/PEDOT:PSS deposited on flat and micro-structured PDMS; (**b**) Schematic representation of the micro-structured PDMS electrodes and (**c**) Optical micrographs of micro-structured PDMS before and after deposition of PEDOT:PSS/AgNWs/PEDOT:PSS and zoomed areas (right) corresponding to high- (top) and low-AgNWs (bottom). Scale bars of left and right images correspond to 2 μm and 500 nm, respectively.

In fact, the assembly of these flexible hybrid electrodes on ITO substrates results in the fabrication of simple solution-processed pressure sensors-like devices (Figure S1, Supplementary Information). However, the results in Table 1 clearly demonstrate that the measured resistance in the flexible electrodes pressure sensors does not depend on the applied pressure. Chou *et al*. demonstrated that PDMS substrates prepared using nano- or micro-structured templates combined with sprayed carbon nanotubes can display such pressure-sensitivity^14^. In their approach, the carbon nanotubes deposited on top of the pyramid-like PDMS (produced by photolithography) were removed using the scotch-tape peeling method, which is a relatively low-control and low-reproducibility technique. As an alternative to photolithography, we generate micro-porous structures using our recently introduced low-cost polymer self-assembly approach^26^. After depositing blends of Poly(3-hexylthiophene-2,5-diyl) (P3HT) and poly(methyl methacrylate) (PMMA) by spin-coating, the PMMA phase is selectively removed to generate micro-porous structures. A detailed analysis of the morphology of these micro-porous structures using atomic force microscopy is presented in the Supplementary Information (Figure S3(a)). Using these micro-porous films as templates, PDMS substrates with micro-islands on their surfaces can be prepared through a scalable solution process (Figure S3(b), Supplementary Information). Note that the devices and results presented here were obtained without using scotch-tape peeling. Furthermore, although a wide dispersion in diameter distribution of the features on PDMS can be found, the heights of these features only display a minor variation and are of approximately 240 nm. The evolution of measured resistance with applied pressure presented in Fig. 1(a) clearly emphasizes that, while flat PDMS electrodes do not exhibit pressure sensitive response, in the micro-structured hybrid films, three distinctive responses can be observed. The resistance measured using micro-structured solution-processed electrodes for applied pressures of 0, 50, 200 and 500 kPa are of over 100 MΩ, 8~10 MΩ, 1~5 kΩ and 480 ~525 Ω, respectively. Consequently, three different levels of pressure can be detected with these hybrid electrodes. Additionally, unlike devices based on evaporated Ag, the solution-processed devices can be fabricated on large scales over 10 × 10 cm^2^ as electrical contact is only generated when pressure is applied. In Ag evaporated devices, the weight of the hybrid electrodes is enough to induce deformation of the PDMS substrate and as the resistivity of evaporated Ag is extremely low, this results in a parasitic electrical contact for device with active areas larger than 0.5 × 0.5 cm^2^. The peculiar properties of micro-structured electrodes in simple pressure sensors can be explained by taking a closer look at the positions of AgNWs aggregates on the PEDOT:PSS covered PDMS substrates (Fig. 1(c)). For flat PDMS substrates, the AgNWs drop-casted from a dilute solution form a dense network which remains relatively flat and with PEDOT:PSS/AgNWs/PEDOT:PSS thicknesses of approximately 200 nm which is slightly below the height of the PDMS micro-islands. After the pre-deposition 30 min ultrasonic treatment to increase the quality of AgNWs dispersion in IPA, the average AgNWs lengths were found to be below 2 μm. When micro-structured PDMS is used, AgNWs which are not covered with PEDOT:PSS are concentrated into regions with a similar arrangement as the micro-islands (Fig. 1(c)). Furthermore, by looking at the areas around the micro-islands, a high density AgNWs network embedded into PEDOT:PSS can be observed. These two regions on top and around the micro-islands will be referred to as high-AgNWs and low-AgNWs, respectively. Note that, AgNWs on flat PDMS substrates are almost entirely embedded in the PEDOT:PSS layer (similarly to low-AgNWs) which is not the case for high-AgNWs located on top of the micro-islands of micro-structured PDMS. In the initial OFF state when no pressure is applied, the high-AgNWs and low-AgNWs are not in electrical contact with each other due to a break in percolation resulting from the difference in heights between the two regions containing AgNWs.

**Figure 2:**
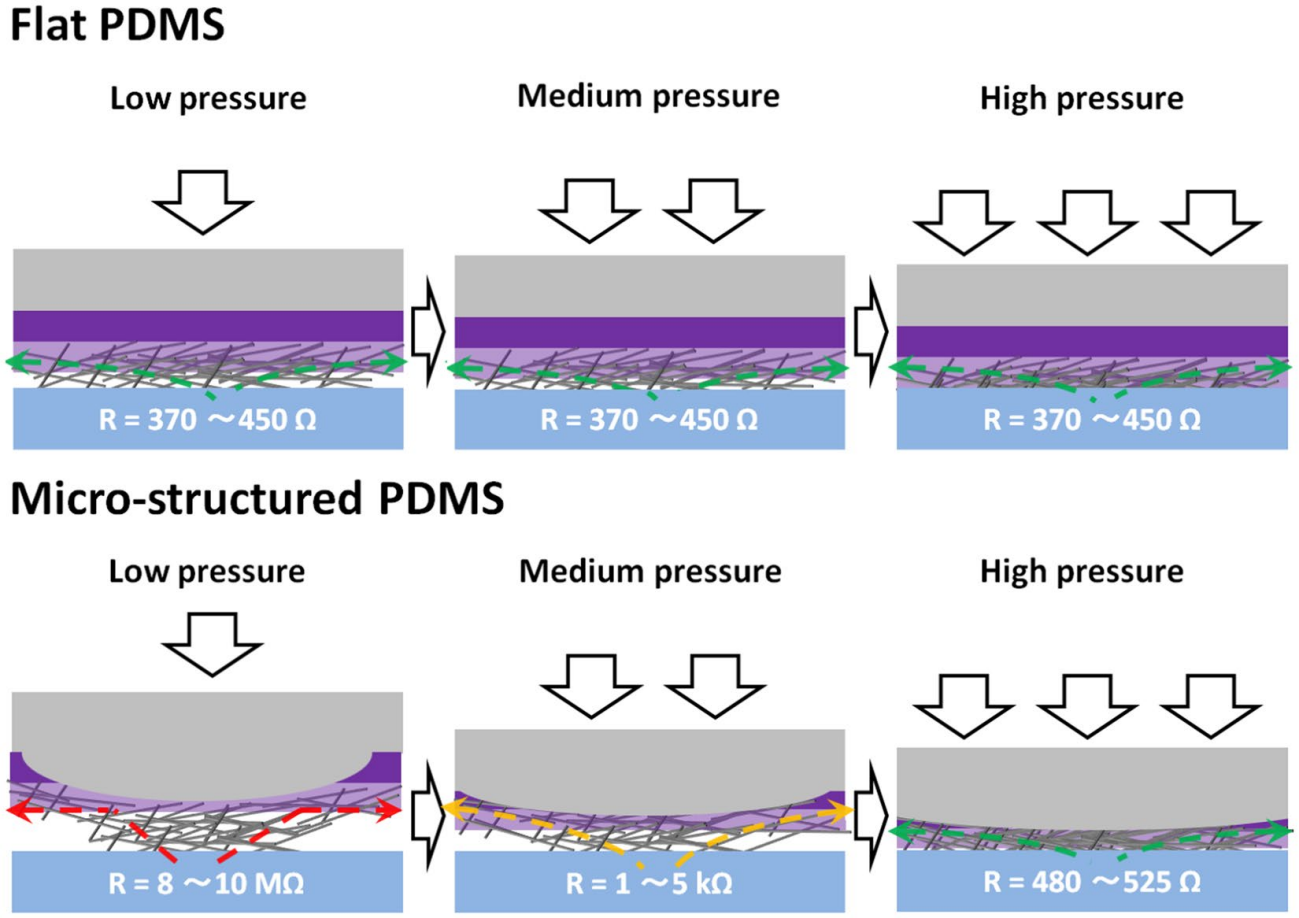
Schematic representation of the working mechanism and percolation paths in flat (top) and micro-structured (bottom) electrode pressure sensors.

In Fig. 2, a comparative schematic representation of the working mechanism of the two pressure sensors is proposed. At low applied pressure, upon deformation of the PDMS micro-islands, the high-AgNWs are compressed between the ITO counter-electrode and the high conductivity PEDOT:PSS/AgNWs hybrid film (low-AgNWs). As the number of high-AgNWs in contact with low-AgNWs is relatively low in these conditions, the probability that charges can efficiently percolate to the low-AgNWs remains relatively low and therefore, high resistances of 8~10 MΩ are measured. By increasing the pressure to 200 kPa, the high-AgNWs are further compressed and generate a much more efficient electrical contact with the low-AgNWs. Consequently, percolation is ensured and charges injected from the ITO counter-electrode will first move along the high-AgNWs and then along the high conductivity continuous network of PEDOT:PSS embedded low-AgNWs. As observed previously (Table 1), uncovered AgNWs have a lower conductivity with respected to PEDOT:PSS covered AgNWs which suggests that the limiting step for percolation corresponds to the charge transport throughout high-AgNWs (not covered with PEDOT:PSS). Surprisingly, the measured resistance for 200 kPa (1~5 kΩ) is higher than the previously measured one for flat substrate uncovered AgNWs (approximately 0.75 kΩ). This small difference could be due to a variation in AgNWs density between high-AgNWs on micro-structured substrates and when deposited on flat PDMS substrates. At high pressures of 500 kPa and over, the ITO counter-electrode comes into direct contact with low-AgNWs and consequently, low resistances (similar to those of PEDOT:PSS embedded AgNWs deposited on flat PDMS) can be measured.

These extremely simple devices have a very promising potential for the development of next-generation electronic devices. In particular, the three specific responses obtained for different ranges of applied pressure can become an interesting tool to fabricate multifunctional devices. In this study, we demonstrate that these hybrid electrodes can also be applied to light-emitting pressure sensor fabrication as discussed in the following section.

### Light-emitting pressure sensors

The device architecture and measurement conditions for light-emitting pressure sensors are presented in Figure S4 (Supplementary Information).Using micro-structured substrates should provide a higher spatial resolution of electrical contact as compared to devices prepared with flat PDMS substrates. In simple pressure sensors where electrical contact is simply translated into variations in resistance, the spatial distribution cannot be adequately visualized. However, by combining an OLED architecture with the flexible hybrid electrodes, we fabricated devices in which pressure distribution can be directly visualized through electroluminescence. The work functions of the two electrodes play an essential role to have efficient devices which is why we used an inverted device architecture in which the solution-processed flexible electrodes can be used as anode. To compare our solution-processed electrodes with evaporated ones, PDMS/evaporated Ag/evaporated molybdenum trioxide (MoO_3_) films were also used as flexible anodes placed on top of ITO/zinc oxide (ZnO)/F8BT substrates (Figure S5, Supplementary Information). MoO_3_ and ZnO play the roles of work function modification as well as charge selective layers in this device configuration. Using the inverted device architecture enables the use of Ag as top anode and increases the device stability as compared to regular device architectures^11^. Additionally, as the flexible anodes are deposited on PDMS, which acts as a barrier for oxygen and moisture, the device stability in air should increase^27^.

Upon application of pressure, the evaporated electrode device switches on and bright green electroluminescence can be observed from F8BT. Due to partial PDMS deformation near the applied pressure contact area and the low resistivity of Ag evaporated on PDMS, a large area around the contact points also switches on. Note that these results are almost independent of the applied pressure (observed even at low pressure) and similar parasitic emission can be observed when using micro-structured PDMS/evaporated Ag electrodes. Additionally, the evaporated electrode is gradually transferred to the F8BT surface after just 5 ON/OFF cycles and the amount of transferred metal increases with the number of repeating cycles (Figure S4, left image). After 20 cycles, the devices cease to function. The values of surface free energies for PDMS, Ag, MoO_3_ and F8BT are 19.9, 1250, 31 and 26 mJ/m^2^, respectively^23, 24, 28, 29^. Consequently, the adhesion of MoO_3_ with F8BT is much stronger than that of Ag and PDMS and that of MoO_3_ with Ag. During the ON state, some MoO_3_ can therefore be easily transferred to the F8BT surface. As established earlier, the stronger adhesion of Ag with MoO_3_ as compared to that of Ag with PDMS also results in a partial transfer of Ag to the F8BT surface together with that of MoO_3_. On the other hand, similarly to the resistive pressure sensors, AgNWs and PEDOT:PSS should exhibit a strong adhesion and consequently, AgNWs should not transfer from the PEDOT:PSS covered PDMS to the F8BT surface. As a result, the devices prepared with solution processed flexible electrodes have a much higher lifetime as compared to the evaporated Ag/MoO_3_ electrodes.

**Figure 3:**
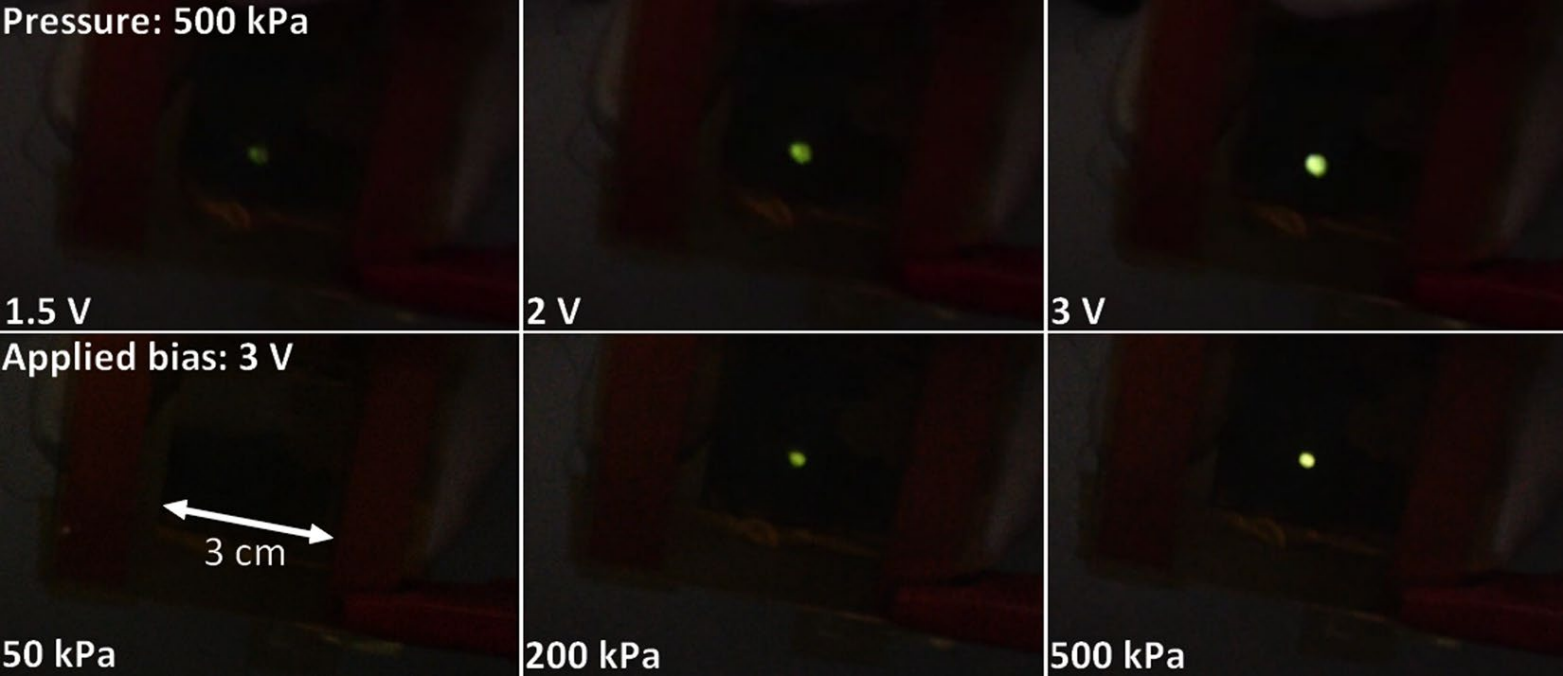
Observed electroluminescence in micro-structured solution-processed light-emitting pressure sensors (top) at constant pressure with increasing bias or (bottom) at constant bias with increasing pressure.

In fact, micro-structured PDMS/PEDOT:PSS/AgNWs/PEDOT:PSS devices only displayed a small increase in turn-on voltages of approximately 200 mV after 300 repeating ON/OFF cycles at 500 kPa. Under these conditions, the electroluminescence from F8BT can already be observed at an applied bias of 1.5 V (Fig. 3). Similarly to conventional OLEDs, the device luminance increases with bias and luminance efficiencies of over 2 cd/A can be measured at higher voltages. Furthermore, in addition to their extremely positive lifetime results, the micro-structured PDMS solution-processed devices demonstrate a pressure-sensitive increase in luminance (Table 2).

**Table 2:** Summary of luminance of micro-structured solution-processed light-emitting sensors.

luminance (cd.m^−2^)	1.5 V	2 V	3 V	50 kPa	200 kPa	500 kPa
constant pressure (500 kPa)	0.1	2.4	18	—	—	—
constant bias (3 V)	—	—	—	0	1.9	17

**Figure 4:**
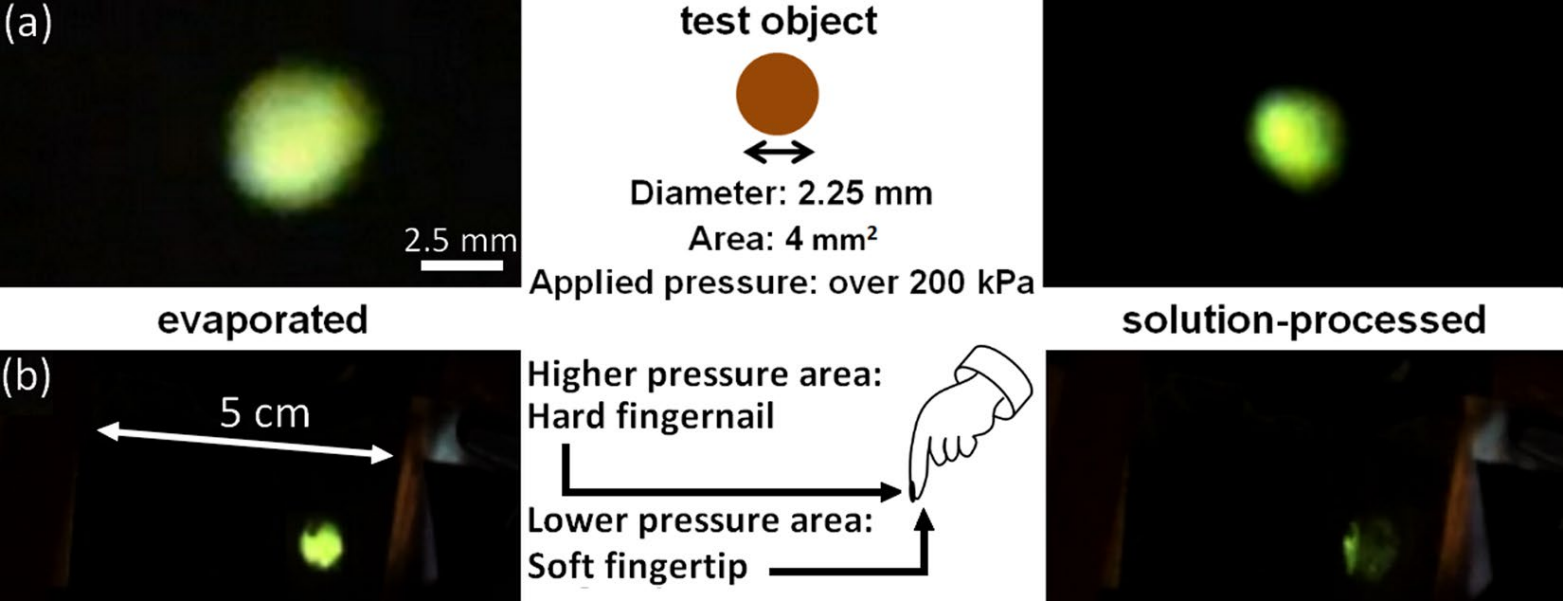
Comparison between the emissions from micro-structured evaporated (left) and solution-processed (right) light-emitting pressure sensors with (**a**) a round test object (optical micrographs) and (**b**) the tip of a finger (photographs of the device).

Unlike the resistive pressure sensors, at low applied pressure (approximately 50 kPa), no emission can be observed. This is easily understood as the resistance through the flexible electrode at low applied pressure is on the MΩ scale. Consequently, holes cannot be injected into the emitting layer and no electroluminescence is observed. By increasing the applied pressure, the second level of electrode resistance is reached (a few kΩ) in which holes are injected into the emitting layer with relatively low injection efficiency and only a low-intensity electroluminescence can be observed. At high applied pressure, the emission becomes much brighter as the resistance of the electrode is reduced by a factor of 2 or more. Note that, even though the difference in resistance between the two types of electrode is of more than two orders of magnitude, the luminance obtained for evaporated electrodes at 3 V is only 1.2 times higher than that obtained with solution processed electrodes. Furthermore, we tested the micro-structured evaporated and solution-processed electrodes when a test object with a round surface (contact area of 4 mm^2^) is pressed against the two devices at a pressure over 500 kPa. The chosen pressure emphasizes the spatial resolution difference between the two device types but similar results are obtained at lower applied pressure (with a lower luminance from the solution-processed devices). In the micrographs presented in Fig. 4(a), when the test object is pressed against the evaporated flexible electrode, an emitting area of 9.1 mm^2^ can be observed. On the other hand, when solution-processed electrodes are employed, the emitting area is reduced to 5.4 mm^2^. Additionally, while the evaporated electrode devices exhibit a relatively homogeneous emission from the whole emitting area, the solution-processed ones display a much more intense emission from the central 4 mm^2^ relative to the border emission.

When pressure is applied to the test object, the PDMS in the adjacent area also undergoes some deformation. Although the deformation should be the same for both evaporated and solution-processed electrodes, the fact that a simple and gentle pressure in the case of evaporated Ag/MoO_3_ on PDMS is sufficient to generate electrical contact is at the origin of the observed large parasitic emission. On the other hand, solution-processed electrodes, which undergo the same deformation, require the application of pressures above 200 kPa to generate electroluminescence. From the emission micrograph, one can clearly observe that such pressure is confined to the PDMS directly adjacent to the area where the 500 kPa pressure is applied. In summary, compared to the evaporated electrodes, the solution-processed electrodes have the potential to confine the unwanted emission to less than 150 microns in the area adjacent to the one where pressure is applied while in the case of evaporated flexible electrodes, the unwanted emission spreads up to 750 microns in the area adjacent to where the test object is positioned.

The second test (Fig. 4(b)) further emphasizes the potential of micro-structured solution-processed electrodes by applying the pressure using the tip of the finger. The tip of the finger is composed of two components, namely, the actual soft tip of the finger, and the hard nail which will apply a higher local pressure to the inorganic elastomer. During this test, the applied pressure was kept as low as possible to observe the contrast between the emission from the high and low pressure areas. When pressure is applied using the tip of the finger to the light-emitting pressure-sensor devices, a round-shaped emission from the soft fingertip can be observed (right side) together with a line-like emission originating from the contact with the fingernail. Although the shapes of the emitting areas observed for the two flexible electrodes are similar, their intensity and, more specifically, the relative intensities of the round- and line-shaped features, are entirely different. The evaporated electrode device displays a similarly bright emission from both areas in contact with the fingertip and fingernail. However, in the devices employing the solution-processed micro-structured electrodes, a bright emission from the fingernail area contrasts the low-intensity electroluminescence from the soft fingertip area.

## Conclusion

In summary, we fabricated two types of low-cost all-solution-processed pressure sensor device, which are referred to as simple and light-emitting pressure sensors, respectively. The flexible electrodes used in these devices consist of a PDMS substrate covered with PEDOT:PSS/AgNWs/PEDOT:PSS trilayers. While the first PEDOT:PSS layer (in contact with PDMS) acts as a compatibilizing interlayer to allow for efficient wetting of the substrate by the AgNWs solution, the second PEDOT:PSS layer, on the other hand, is used to partially embed the AgNWs into a hybrid PEDOT:PSS/AgNWs film with high conductivities and the adequate work function to be used as anode in inverted OLED architectures. Furthermore, these flexible devices with resistance values around 400 Ω have a transmittance of 80% spreading throughout the visible range and can therefore be considered as potential candidates for low-cost fabrication of touch panels. The integration of micro-structures into the PDMS substrates results in an applied pressure-sensitive response. The measured resistances in these sensors when 50,200 and 500 kPa are applied is in the MΩ, kΩ and hundreds of Ω ranges, respectively.

Light-emitting pressure sensors are fabricated using an inverted OLED architecture combined with a top flexible electrode. The micro-structured solution-processed electrode devices not only exhibit pressure-sensitive luminance but also a higher spatial resolution as compared to the evaporated electrode devices. Additionally, due to a stronger adhesion of the PEDOT:PSS embedded AgNWs to the PDMS substrate and the increased flexibility of this hybrid multilayer with respect to evaporated Ag, the number of ON/OFF cycles is increased from 50 for the evaporated electrodes to over 300 cycles for the solution-processed ones. Consequently, the devices presented here open the path to the fabrication of low-cost next-generation technologies such as enhanced optical devices for fingerprint visualization, wearable electronics or on-board light-communication devices and electronic skin.

## Methods

### Flexible electrode fabrication and characterization

PDMS elastomer (Sylgard 184) was purchased from Dow Corning. To fabricate the flexible substrates, the PDMS precursor was mixed with the curing agent (10:1 weight ratio) and stirred using a glass pipette. After degassing the mixture to remove air bubbles formed during stirring, standard glass Petri dishes with polished surface were used for the mixture deposition which is cured at 80 °C for at least two hours. The thickness of the PDMS substrates (approximately 3 mm) was controlled by the volume of mixture deposited on the cleaned Petri dishes. PDMS substrates are treated for 30 min in a plasma generated under a pressure of 200~600 mTorr with air prior to metal/semiconductor layer deposition.

PEDOT:PSS (Clevios^TM^ PH1000) was purchased from Heraus. Triton^TM^ × 100 (0.5 vol%, from Sigma-Aldrich) and DMSO (5 vol%, from Sigma-Aldrich) were added to the PEDOT:PSS dispersion to ensure proper wetting and increase its conductivity, respectively. The first PEDOT:PSS on PDMS was deposited by spin-coating the mixture at 4000 rpm for 30 s resulting in PEDOT:PSS film thicknesses of approximately 35 nm. The AgNWs layer was deposited by drop-casting a diluted suspension (Sigma-Aldrich, 1 mg/ml) on the PEDOT:PSS covered PDMS substrate. After complete drying of the drop-casted AgNWs (approximately 60 min), a second PEDOT:PSS layer was deposited by spin-coating at 1000 rpm for 60 s in the case of the trilayers. The thickness of this second PEDOT:PSS layer was approximately 120 nm. After all layers are deposited, the flexible electrodes were annealed at 150 °C for 15 min prior to characterization.

For evaporated electrode fabrication, the PDMS substrates were placed in a vacuum chamber, followed by thermal evaporation of Ag (120 nm) and MoO_3_ (20 nm) at respective speeds of 3 and 0.5 Å.s^−1^.

The transmittance spectra were measured using a UV-Vis spectrophotometer (JASCO V-730) with air as a reference. Optical micrographs were obtained using a DSX510 microscope (Olympus) in bright-field mode.

### Self-assembled micro-structured template and micro-structured flexible electrode fabrication

Self-assembled micro-structured polymer templates were prepared following a method published previously^26^. P3HT (Mw~200,000, Merck) and PMMA (Mw~15,000, Sigma-Aldrich) are mixed in chlorobenzene at a 1:1 ratio and with a total polymer concentration of 30 mg.ml^−1^. The blend is then spin-coated at 1000 rpm for 60 s on clean glass substrates and the PMMA phase is selectively removed using acetone generating micro-porous structures with average pore diameters and depths of 1.65 and 0.25 μm, respectively. The PDMS precursor and curing agent mixture is then poured on the micro-structured templates similarly to the Petri dishes for flat PDMS substrate fabrication. Solution-processed and thermally evaporated electrodes are deposited as described in the previous section.

### Device fabrication and characterization

Resistive pressure sensors are fabricated following the simple procedure described hereafter: ITO covered glass substrates (sheet resistance below 20 Ω/sq) are cleaned using acetone, surfactant, deionized water and IPA in an ultrasonic bath followed by exposure to IPA vapor for 3 min. After placing the insulating tape, conductive copper tape and silver paste as described in Figure S1, the flexible electrodes are placed on top of the substrates with the metals and semiconducting layers facing downwards. The devices are tested under various applied pressure and the resistance variations are measured using an Agilent U1241B tester.

For light-emitting pressure sensors, ZnO layers were prepared on plasma treated cleaned ITO covered glass substrates. A precursor mixture composed of 500 mg ZnOAc.H2O and 101.4 mg 2-aminoethanol in 5 ml of 2-methoxyethanol was stirred for 3 hours at room temperature. Once a homogenous solution was obtained, the mixture was spin-coated on the substrates at a speed of 3000 rpm for 30 s. The resulting layers were annealed at 200 °C for 30 min followed by slow cooling until room temperature. Then, a 100-nm thick layer of F8BT (Sigma-aldrich) was deposited by spin-coating a solution in chlorobenzene at 1500 rpm for 60 s. After placing the insulating tape, conductive copper tape and silver paste as described in Figure S4, the flexible electrodes are placed on top of the substrates with the metals and semiconducting layers facing downwards. The devices were characterized by applying pressure on the flexible electrodes while controlling applied voltages with a Keithley 2401 sourcemeter. The variation in emitted light-intensity and spatial distribution was measured using simple photographs or an optical microscope (AS Tools) equipped with a USB camera. The luminance measurements were performed using a Topcon BM-9A luminance meter.

## Electronic supplementary material

Supplementary Information

## References

[1] Xu R-P, Li Y-Q, Tang J-X (2016). Recent advances in flexible organic light-emitting diodes. J. Mater. Chem. C.

[2] Yi HT, Payne MM, Anthony JE, Podzorov V (2012). Ultra-flexible solution-processed organic field-effect transistors. Nat. Commun..

[3] Rao S, Morankar A, Verma H, Goswami P (2016). Emerging Photovoltaics: Organic, Copper Zinc Tin Sulphide, and Perovskite-Based Solar Cells. J. Appl. Chem..

[4] Sirringhaus H (2014). 25th Anniversary Article: Organic Field-Effect Transistors: The Path Beyond Amorphous Silicon. Adv. Mater..

[5] Aguirre CM (2016). Carbon nanotube sheets as electrodes in organic light-emitting diodes. Appl. Phys. Lett..

[6] Wu X (2012). Highly efficient flexible organic light-emitting devices utilizing F4-TCNQ/m-MTDATA multiple quantum well structures. J. Lumin..

[7] Hast J (2013). 18.1: Invited Paper: Roll-to-Roll Manufacturing of Printed OLEDs. SID Symp. Dig. Tech..

[8] Kobayashi T, Yokoyama T, Utsumi Y, Kanematsu H, Masuda T (2013). Study on Evaluation Methods for Mechanical Properties of Organic Semiconductor Materials. J. Phys.: Conf. Ser..

[9] Printz AD, Lipomi DJ (2016). Competition between deformability and charge transport in semiconducting polymers for flexible and stretchable electronics. Appl. Phys. Rev..

[10] Kabra D, Lu LP, Song MH, Snaith HJ, Friend RH (2010). Efficient Single-Layer Polymer Light-Emitting Diodes. Adv. Mater..

[11] Bolink H, Coronado E, Orozco J, Sessolo M (2009). Efficient Polymer Light-Emitting Diode Using Air-Stable Metal Oxides as Electrodes. Adv. Mater..

[12] Lu L, Kabra D, Friend RH (2012). Barium Hydroxide as an Interlayer Between Zinc Oxide and a Luminescent Conjugated Polymer for Light-Emitting Diodes. Adv. Funct. Mater..

[13] Wang C (2013). User-interactive electronic skin for instantaneous pressure visualization. Nat. Mater..

[14] Chou H-H (2015). A chameleon-inspired stretchable electronic skin with interactive colour changing controlled by tactile sensing. Nat. Commun..

[15] Huang G-W, Xiao H-M, Fu S-Y (2015). Wearable Electronics of Silver-Nanowire/Poly(dimethylsiloxane) Nanocomposite for Smart Clothing. Sci. Rep..

[16] Chen S, Cui Q, Guo X (2015). Annealing-Free Solution-Processed Silver Nanowire-Polymer Composite Transparent Electrodes and Flexible Device Applications. IEEE Trans Nanotechnol..

[17] Qingqing Y, Jinliang Y, Delan M (2015). Transparent and conductive PEDOT:PSS/Ag NW/PEDOT:PSS hybrid films prepared by spin-coating at room temperature. J. Semicond..

[18] Eom J, Lee W, Kim Y-H (2016). Textile-based wearable sensors using metal-nanowire embedded conductive fibers. IEEE Sensors.

[19] Vohra V, Anzai T, Inaba S, Porzio W, Barba L (2016). Transfer-printing of active layers to achieve high quality interfaces in sequentially deposited multilayer inverted polymer solar cells fabricated in air. Sci. Tech. Adv. Mater..

[20] Pasha A, Roy AS, Murugendrappa MV, Al-Hartomy OA, Khasim S (2016). Conductivity and dielectric properties of PEDOT-PSS doped DMSO nano composite thin films. J. Mater. Sci: Mater. Electron..

[21] Lee I, Kim GW, Yang M, Kim T-S (2016). Simultaneously Enhancing the Cohesion and Electrical Conductivity of PEDOT:PSS Conductive Polymer Films using DMSO Additives. ACS Appl. Mater. Interf..

[22] Kim JS, Friend RH, Cacialli F (1999). Surface energy and polarity of treated indium–tin–oxide anodes for polymer light-emitting diodes studied by contact-angle measurements. J. Appl. Phys..

[23] Vitos L, Ruban AV, Skriver HL, Kollár J (1998). The surface energy of metals. Surf. Sci..

[24] M.J. Owen. First international congress on adhesion science and technology. (VSP BV, 1998).

[25] Petrosino M, Rubino A (2012). The effect of the PEDOT:PSS surface energy on the interface potential barrier. Synt Met..

[26] Vohra V (2016). Investigating phase separation and structural coloration of self-assembled ternary polymer thin films. Appl. Phys. Lett..

[27] Han J-M, Han J-W, Chun J-Y, Ok C-H, Seo D-S (2008). Novel Encapsulation Method for Flexible Organic Light-Emitting Diodes using Poly(dimethylsiloxane). Jpn. J. Appl. Phys..

[28] Papakondylis A, Sautet P (1996). Ab Initio Study of the Structure of the α-MoO3 Solid and Study of the Adsorption of H2O and CO Molecules on Its (100) Surface. J. Phys. Chem..

[29] Cataldo S, Sartorio C, Giannazzo F, Scandurra A, Pignataro B (2014). Self-Organization and nanostructural control in thin film Heterojunctions. Nanoscale.

