# Supplementary material for: All solution-processed micro-structured flexible electrodes for low-cost light-emitting pressure sensors fabrication
Source: Sci Rep. 2017 Jul 31;7:6921. doi: 10.1038/s41598-017-07284-8 (PMC5537286; doi:10.1038/s41598-017-07284-8)
Supplement: Supplementary file 1 — Supplementary Information [file 41598_2017_7284_MOESM1_ESM.pdf]

Supplementary information

All solution-processed micro-structured flexible electrodes for low-cost light-emitting pressure sensors fabrication

Rie SHIMOTSU, Takahiro TAKUMI, and Varun VOHRA*
Department of Engineering Science, University of Electro-Communications
1-5-1 Chofugaoka, Chofu, Tokyo, 182-8585, Japan
*E-mail: varun.vohra@uec.ac.jp

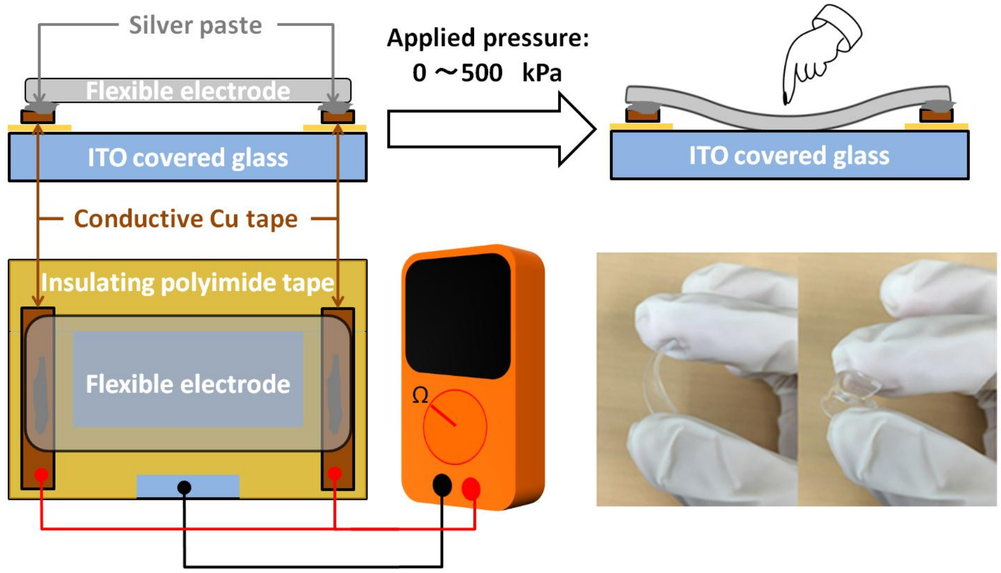

Figure S1. Device architecture and testing conditions of flexible hybrid electrodes at various pressures and photographs of bent PDMS substrates.

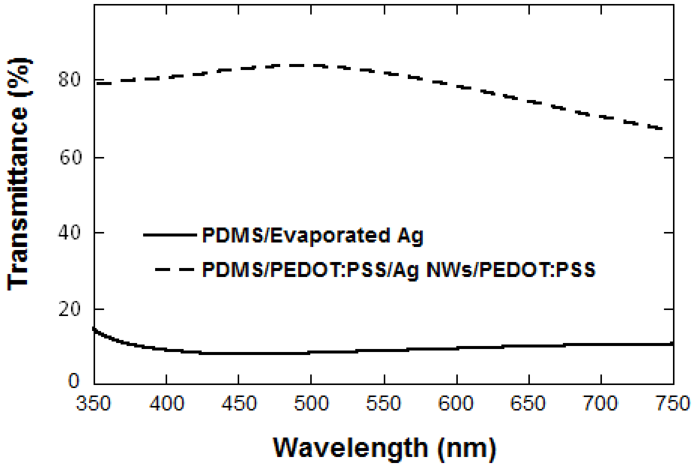

Figure S2. Comparative transmittance of evaporated and solvent-processed hybrid flexible electrodes.

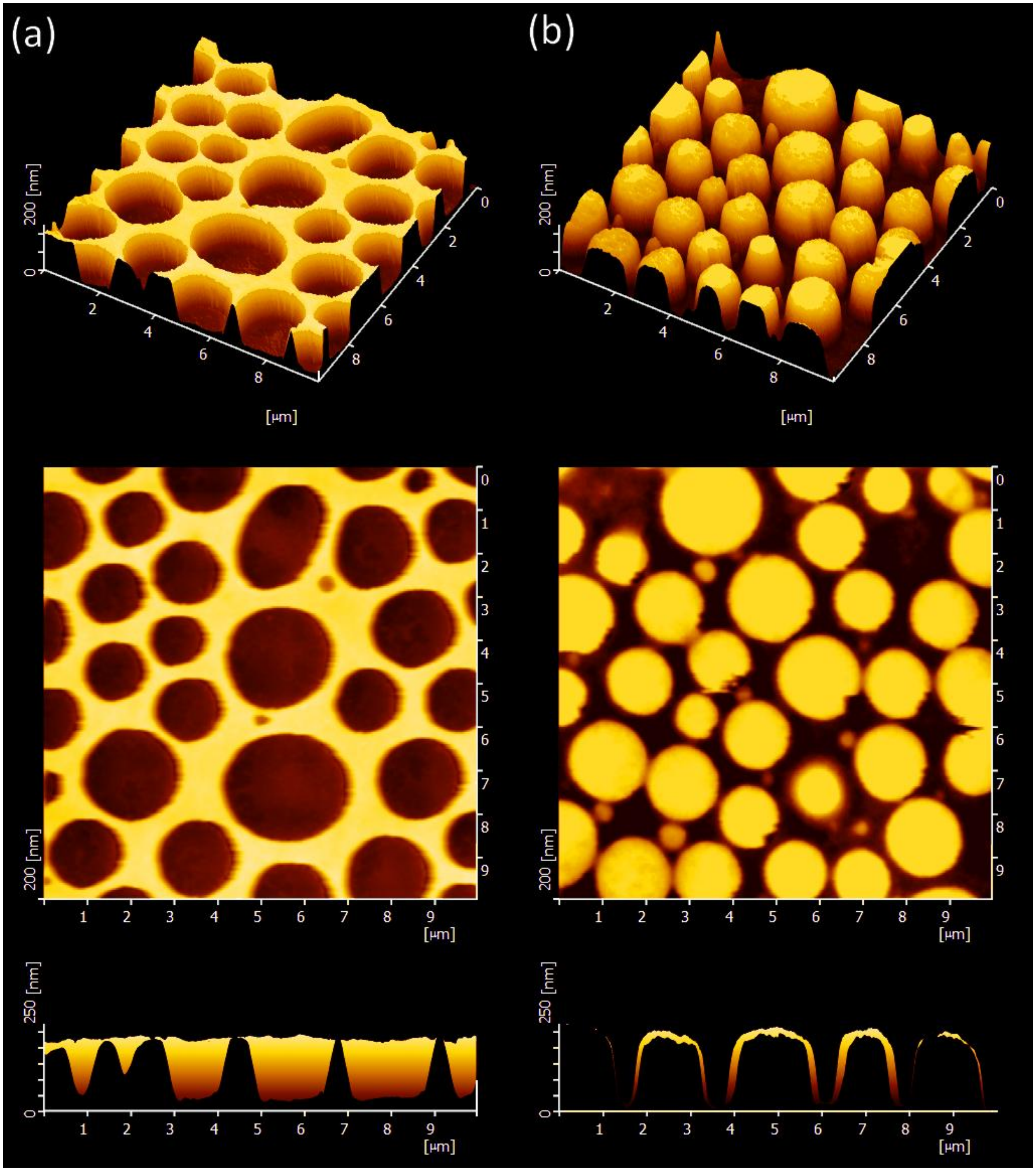

Figure S3. Inclined, top and side views of three-dimensional atomic force images measured on (a) self-assembled micro-porous polymer templates and (b) micro-structured PDMS substrates.

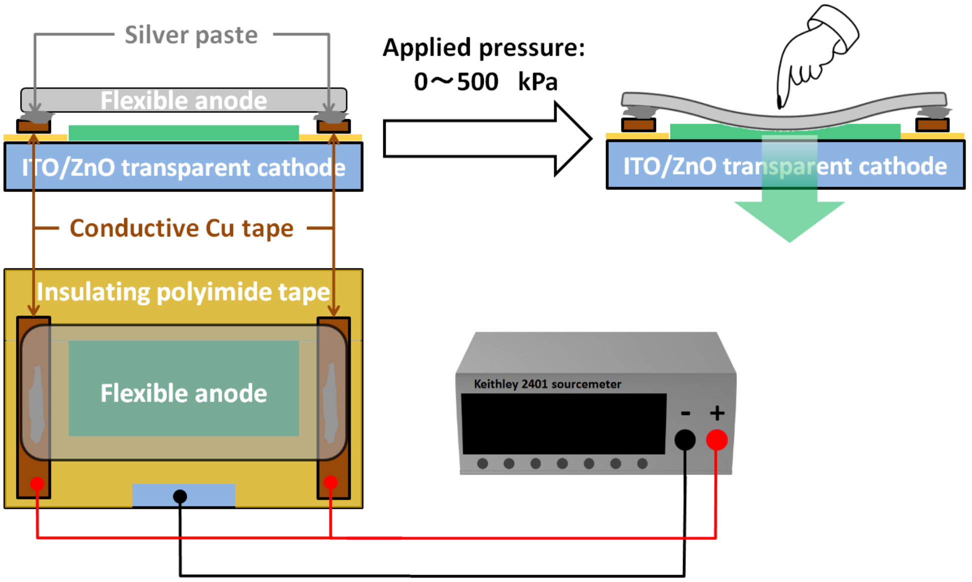

Figure S4. Device architecture and testing conditions of flexible hybrid electrodes light-emitting pressure sensors.

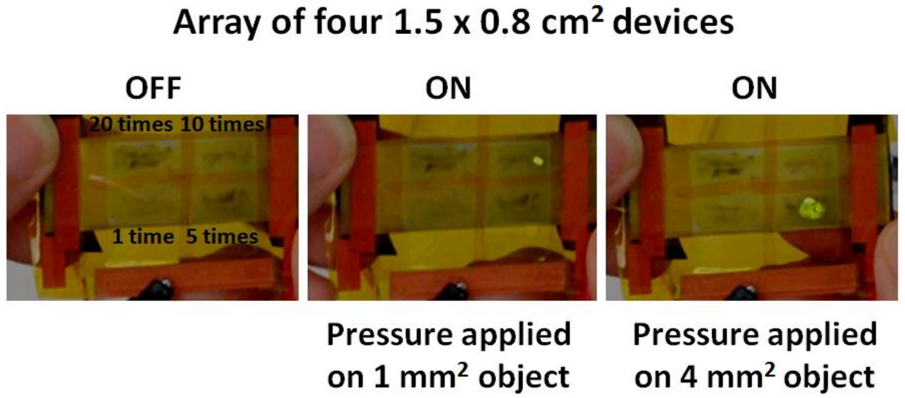

Figure S5. OFF and ON states of the light-emitting devices fabricated using Ag/MoO_3 deposited on PDMS by thermal evaporation.
